# Large-scale mutational analysis of wheat powdery mildew resistance gene *Pm21*

**DOI:** 10.3389/fpls.2022.988641

**Published:** 2022-08-09

**Authors:** Huagang He, Rui Guo, Anli Gao, Zhaozhao Chen, Renkang Liu, Tianlei Liu, Xusen Kang, Shanying Zhu

**Affiliations:** ^1^School of Life Sciences, Jiangsu University, Zhenjiang, China; ^2^School of Food and Biological Engineering, Jiangsu University, Zhenjiang, China; ^3^School of Life Sciences, Henan University, Kaifeng, China; ^4^School of Environment, Jiangsu University, Zhenjiang, China

**Keywords:** wheat powdery mildew, *Pm21*, nucleotide-binding leucine-rich repeat receptor (NLR), mutational analysis, mutation hotspot, dominant-negative effect

## Abstract

Wheat powdery mildew is a devastating disease leading to severe yield loss. The powdery mildew resistance gene *Pm21*, encoding a nucleotide-binding leucine-rich repeat receptor (NLR) protein, confers broad-spectrum resistance to powdery mildew and has great potential for controlling this disease. In this study, a large-scale mutagenesis was conducted on wheat cultivar (cv.) Yangmai 18 carrying *Pm21*. As a result, a total of 113 independent mutant lines susceptible to powdery mildew were obtained, among which, only one lost the whole *Pm21* locus and the other 112 harbored one- (107) or two-base (5) mutations in the encoding region of *Pm21*. From the 107 susceptible mutants containing one-base change, we found that 25 resulted in premature stop codons leading to truncated proteins and 82 led to amino acid changes involving in 59 functional sites. We determined the mutations per one hundred amino acids (MPHA) indexes of different domains, motifs, and non-domain and non-motif regions of PM21 protein and found that the loss-of-function mutations occurred in a tendentious means. We also observed a new mutation hotspot that was closely linked to RNBS-D motif of the NB-ARC domain and relatively conserved in different NLRs of wheat crops. In addition, we crossed all the susceptible mutants with Yangmai 18 carrying wild-type *Pm21*, subsequently phenotyped their F_1_ plants and revealed that the variant E44K in the coiled-coil (CC) domain could lead to dominant-negative effect. This study revealed key functional sites of PM21 and their distribution characteristics, which would contribute to understanding the relationship of resistance and structure of *Pm21*-encoded NLR.

## Introduction

Plants have developed a fine two-layered innate immune system to defense pathogens during long-time evolution. The first layer is pattern-triggered immunity (PTI) that is promoted *via* an activation of plasma membrane-resident pattern recognition receptors (PRRs) by perception of pathogen-associated molecular patterns (PAMPs). The second layer is effector-triggered immunity (ETI) that is elicited by pathogen effectors directly or indirectly recognized by intracellular nucleotide-binding leucine-rich repeat receptors (NLRs), commonly accompanied with hypersensitivity reaction (HR) leading to host cell death at the local site invaded by pathogens. It is considered that PTI contributes to basal defense and ETI provides race-specific resistance to pathogens (Dangl et al., 2013; Sun et al., 2020).

A typical plant NLR protein contains an N-terminal domain, a central nucleotide-binding (NB) domain, and a C-terminal leucine-rich repeat (LRR) domain. The variable N-terminus of NLR usually includes a Toll-interleukin 1 receptor (TIR) domain or a coiled-coil (CC) domain. Correspondingly, NLRs are classified into two types, TIR-type NLRs (TIR-NLRs) and CC-type NLRs (CC-NLRs) (Cui et al., 2015; Cesari, 2018). The central NB domain is highly conserved that works as a molecular switch to alter “off” and “on” state of NLR *via* binding adenosine diphosphate (ADP) and adenosine triphosphate (ATP) (Bernoux et al., 2016). The LRR domain participates in direct or indirect perception of effectors delivered into host cell by pathogens. The CC or TIR domains of many NLRs perform signal transduction and trigger cell death *via* homodimerization (Bernoux et al., 2011; Bai et al., 2012). Recently, it was found that TIR domain is a NADase that can cleave NAD^+^ into nicotinamide and ADP-ribose and transduce signaling into cell death response (Horsefield et al., 2019; Wan et al., 2019). In a plant NLR protein, the above three different domains may interact intramolecularly each other, which can maintain NLR under the status of autoinhibition (Moffett et al., 2002; Wang et al., 2019a,b).

Wheat powdery mildew, caused by the biotrophic fungus *Blumeria graminis* f. sp. *tritici* (DC.) Speer (*Bgt*), is a devastating disease leading to severe yield losses. Up to date, over 80 formally designated wheat powdery mildew resistance genes/alleles have been characterized from wheat and its cultivated and wild relatives. Among them, several genes have been cloned (He et al., 2021a,b). *Pm21*, originated from wheat wild relative *Dasypyrum villosum* (L.) Candargy, confers broad-spectrum resistance to *Bgt* pathogen and displays a great value for controlling wheat powdery mildew. Recently, using a map-based cloning strategy, *Pm21* has been cloned that encodes a typical NLR protein (GenBank accession number: MF370199) (He et al., 2018). Transient expression assay suggests that PM21 protein is autoinhibited *via* intramolecular interactions of its different domains (Gao et al., 2020). Evolutionary analysis, based on a series of *Pm21* alleles isolated from different accessions of *D. villosum*, reveals that the LRR domain has higher genetic diversity than other domains and the solvent-exposed LRR residues have been undergone diversifying selection (dN/dS = 3.20), which is similar to the race-specific powdery mildew resistance genes *Pm3* from wheat and *Mla* from barley (He et al., 2020). In general, we know less about the characteristics of *Pm21* so far.

Mutational analysis is a powerful approach for uncovering structure and function of genes of interest. In the past years, several large-scale mutagenesis researches have been conducted on plant NLR genes, such as *Arabidopsis RPS2* (Tao et al., 2000) and *RPM1* (Tornero et al., 2002), both of which confer resistance against *Pseudomonas syringae*, and tobacco mosaic virus resistance gene *N* (Dinesh-Kumar et al., 2000). However, there is no systemic mutagenesis research focused on an NLR gene-derived wheat crops. Here, we report over a hundred *Bgt*-susceptible mutants of common wheat cv. Yangmai 18 carrying *Pm21 via* ethyl methanesulfonate (EMS) mutagenesis and identify dozens of mutated sites that lead to lose *Pm21* resistance. These mutations will contribute to understanding the molecular basis of PM21 protein.

## Materials and methods

### Plant materials and growth conditions

Wheat cv. Yangmai 18 is a common wheat-*D. villosum* translocation line that carries *Pm21* and confers effective resistance to powdery mildew. Yangmai 23, a susceptible wheat cv., was used as a negative control and a host for reproducing asexual spores of *Bgt*. Wheat plants were grown in the field or in a greenhouse.

### Ethyl methanesulfonate treatment

About 8,000 seeds of Yangmai 18 carrying *Pm21* were soaked with 0.05 M phosphate buffer solution (PBS, pH 7.0) for 4 h. The seeds were then soaked with 0.8% EMS diluted in 0.05 M PBS (pH 7.0) and shook at 20°C for 12 h. The seeds treated with EMS were washed with running water for 4 h and then placed at 4°C for 3 days. After germination, the seeds were sowed in the field for generation. All seeds of each single plant were harvested as an M_2_ family.

### Preliminary screening of mutants susceptible to powdery mildew

About 100 seeds of each M_2_ family were sowed in a pot. The plants at one-leaf stage were inoculated with *Bgt* isolate BgtYZ01 in a greenhouse with LED light under long-day condition (16-h light/8–h dark) at 24 ± 2°C. The powdery mildew responses were assessed at 7 days after inoculation. The susceptible individuals were kept and planted in the field. The responses of the harvested susceptible mutants were re-assessed with the same *Bgt* isolate at the seedling stage. Infection types (IT) were scored according to a 0–4 scale (He et al., 2021b).

### Allelism test of candidate susceptible mutants

Each of the obtained candidate susceptible mutants was crossed with the mutant line Y18-S7, who carries a mutation site at position 834 (G to A) of *Pm21* leading to a premature stop codon and be completely susceptible to isolate BgtYZ01 at whole growth stages (He et al., 2018). The powdery mildew responses of the derived F_1_ plants were detected with BgtYZ01 again to understand whether the mutation leading to susceptibility occurs in *Pm21*.

### Detection of mutation sites

Total RNAs were extracted from leaves of each susceptible mutant using the TRIzol reagent (Life Technologies, Carlsbad, CA, United States), and 2 μg of total RNA was used to synthesize the first-strand cDNA using a PrimeScript™ II 1st Strand cDNA Synthesis Kit (TaKaRa, Shiga, Japan). The gene *Pm21* was PCR-amplified from the cDNA using the high fidelity PrimeSTAR Max Premix (TaKaRa, Shiga, Japan) and the primer pair (forward primer: 5′-TTACCCGGGCTCACCCGTTGGACTTGGACT-3′; reverse primer: 5′-CCCACTAGTCTCTCTTCGTTACATAATGTAGTG CCT-3′). PCR products were digested with *Sma*I and *Spe*I, inserted into the vector pAHC25-MCS1 (He et al., 2018) and sequenced. Each mutation site was further verified by sequencing the independent PCR products.

### Annotation of domains and motifs of PM21 protein and statistical analysis of mutation frequency

In PM21 protein, the CC, NB-ARC, and LRR domains and LRR motifs were annotated based on our previous work (Gao et al., 2020; He et al., 2020). The nT motif in the CC domain was characterized as described by Bai et al. (2002). The motifs in the NB-ARC domain were identified as described by Meyers et al. (1999) and Dilbirligi and Gill (2003). Mutation frequency in full-length protein, different domains, and motifs was calculated by the index of mutations per one hundred amino acids, simply named MPHA index. Sequence logos were created by the WebLogo tool (Crooks et al., 2004) using different NLR proteins in wheat crops.

### Screening and verification of susceptible mutants with dominant-negative phenotype

Yangmai 18 carrying wild-type *Pm21* was used as the female parent to cross, respectively, with different susceptible mutants. The powdery mildew responses of the generated F_1_ plants were assessed by inoculation with BgtYZ01. The phenotypes of the F_2_ plants derived from the susceptible F_1_ were further tested with the same isolate. Chi-squared (χ^2^) test was used to determine the goodness-of-fit of the observed segregation ratio to theoretical Mendelian ratio.

## Results

### A collection of loss-of-function of *Pm21* consists of 113 independent mutants

**FIGURE 1:**
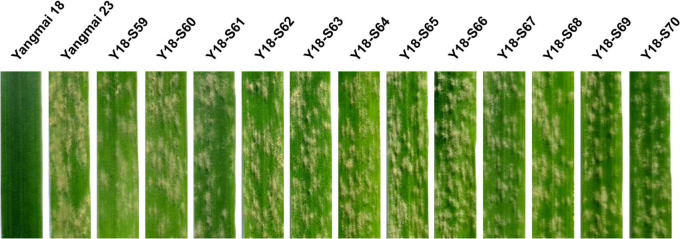
Powdery mildew responses of 12 representative susceptible mutants of Yangmai 18. Responses of the mutant lines Y18-S59∼Y18-S70 to *Bgt* isolate BgtYZ01 were assessed at one-leaf stage. Wheat cv. Yangmai 18 carrying wild-type *Pm21* and cv. Yangmai 23 were used as the resistant and susceptible controls, respectively.

Ethyl methanesulfonate -induced mutagenesis was conducted on Yangmai 18 carrying *Pm21*. From derived 4,462 M_2_ families, a total of 116 candidate mutants susceptible to *Bgt* isolate BgtYZ01 were identified. We also investigated the phenotypes of M_3_ plants of the candidate mutants at the seedling stage and confirmed that all of them lost resistance completely. To exclude loss-of-function mutants caused by non-target mutations, all candidate mutants were crossed with Y18-S7 that had a premature stop codon (G834A) in *Pm21* leading to encode a 280-aa truncated protein and completely susceptible to BgtYZ01 at whole growth stages (He et al., 2018). The generated F_1_ plants derived from 113 crosses were all susceptible whereas those from 3 crosses were resistant, suggesting that the susceptibility of the corresponding 113 susceptible mutants was truly caused by mutated *Pm21*. Of the 113 susceptible mutants, 58 were reported in our previous work (He et al., 2017, 2018) and 55 were newly obtained in this study (Figure 1 and Supplementary Table 1). Except Y18-S6 that had a deletion of the *Pm21* locus, each of the other 112 contained one- (107) or two-base (5) changes in the encoding sequence of *Pm21*. Due to that it was unclear which change(s) led to loss the function in the 5 mutants harbored two mutation sites in *Pm21*, only the 107 susceptible mutants contained one mutation site were used for further statistical analysis.

**FIGURE 2:**
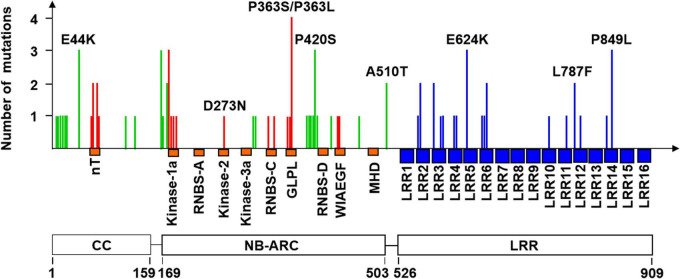
Frequency and distribution of 59 unique mutations leading to amino acid changes. The locations of the coiled-coil (CC), NB-ARC, and leucine-rich repeat (LRR) domains of PM21 are shown at bottom. The known conserved motifs (orange) of the CC and NB-ARC domains and 16 leucine-rich repeat motifs (blue) in the LRR domain are shown below the horizontal axis. The mutations in the motifs and non-motif regions of the CC and NB-ARC domains are shown as vertical red and green lines, respectively. The mutations in the LRR domain are shown as blue lines. A total of eight representative mutations are marked in the figure. More details of all mutation sites are listed in Supplementary Table 1.

In general, of the 107 mutation sites, 59 and 47 were involved in the transition of G to A (55.2%) and C to T (43.9%), respectively, and only one (Y18-S113) was involved in the transversion of A to T (0.9%). Of the 107 mutations of *Pm21*, 25 resulted in premature stop codons leading to truncated proteins ranged from 178 to 843 aa. Compared with full-length PM21 protein that contains 16 LRR motifs as described in our previous work (He et al., 2020), the largest truncated protein (843 aa in Y18-S47) retained 13 LRR motifs, suggesting the importance of the last three LRR motifs (LRR14–LRR16) for resistance. Besides, 82 mutations of *Pm21* each led to a single amino acid change, of which, 59 mutations were unique and the other 23 were repetitive (Figure 2 and Supplementary Table 1).

### Loss-of-function mutations of *Pm21* occur in a tendentious means

In further study, the distribution characteristics of the above 59 distinctive mutations that led to single amino acid changes were analyzed using the index mutations per one hundred amino acids (MPHA). The data showed that the MPHA indexes of the CC (10.1) and NB-ARC (7.2) domains were higher than that of the full-length PM21 (6.5), whereas the MPHA index of the LRR domain (4.4) was lower than that of the full-length PM21. In addition, the MPHA indexes of the Linker 1 (the linker between the CC and NB-ARC domains) and the Linker 2 (the linker between the NB-ARC and LRR domains) were 11.1 and 4.5, which were similar to those of the CC and LRR domains, respectively (Table 1). It was indicated that the two linker regions also play the critical roles in powdery mildew resistance.

**TABLE 1:** Distribution and frequency of loss-of-function mutations altering amino acids in different domains and motifs of PM21.

Domain or motif	Position	Number of mutants	Number of mutations	MPHA index
FL	1–909	82	59	6.5
CC domain	1–159	21	16	10.1
Non-motif region	–	15	12	8.6
nT motif	68–86	6	4	21.1
Linker 1	160–168	3	1	11.1
NB-ARC domain	169–503	31	24	7.2
Non-motif region	–	8	7	2.5
Motif region	–	25	18	19.6
Kinase-1a (P-loop)	196–203	6	4	50.0
RNBS-A	215–227	0	0	0
Kinase-2	269–276	1	1	12.5
Kinase-3a (RNBS-C)	294–302	0	0	0
RNBS-C	319–338	2	2	10.0
GLPL	359–366	6	4	57.1
LPLHLRP	414–420	7	5	71.4
RNBS-D	421–431	1	1	9.1
WIAEGF	443–448	0	0	0
MHD	489–491	0	0	0
Linker 2	504–525	2	1	4.5
LRR domain	526–909	25	17	4.4
LRR1	526–546	0	0	0
LRR2	547–570	3	2	8.3
LRR3	571–596	4	3	11.5
LRR4	597–619	2	2	8.7
LRR5	620–641	3	1	4.8
LRR6	642–662	4	3	14.3
LRR7	663–685	0	0	0
LRR8	686–712	0	0	0
LRR9	713–739	0	0	0
LRR10	740–762	1	1	4.3
LRR11	763–786	1	1	4.2
LRR12	787–809	3	2	8.7
LRR13	810–832	0	0	0
LRR14	833–857	4	2	8.0
LRR15	858–887	0	0	0
LRR16	888–909	0	0	0

MPHA index, number of mutations per one hundred amino acids; FL, full-length protein; Linker 1, the linker between the CC and NB-ARC domains; Linker 2, the linker between the NB-ARC and LRR domains.

**FIGURE 3:**
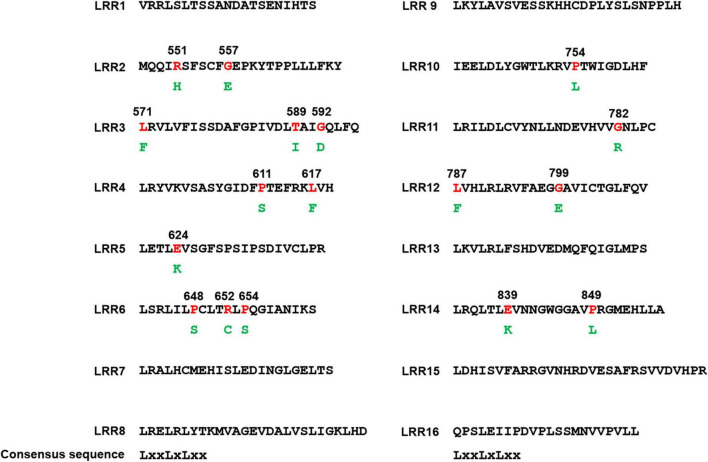
Mutation sites in the leucine-rich repeat (LRR) domain. The LRR domain of PM21 can be divided into 16 LRR motifs and their consensus sequence LxxLxLxx is given at the bottom of this figure. Substituted amino acids (green) and their positions are shown in the below and the above of the mutated ones (red), respectively.

In the CC domain, the MPHA index of the nT motif (21.1) was obviously higher than that of non-motif region (8.6). In the NB-ARC domain, the MPHA index of motif region (19.6) was significantly higher than that of non-motif region (2.5). Strikingly, the MPHA indexes of Kinase-1a (also called P-loop or Walker A) and GLPL motifs reached 50.0 and 57.1, respectively, which were significantly higher than those of other motifs reported previously. Additionally, no mutation was observed in the motifs RNBS-A, Kinase-3a (also named RNBS-B), WIAEGF, and MHD. All the mutation sites in conserved motifs in the CC and NB-ARC domains are listed in Table 2. In the LRR domain, the MPHA indexes of the motifs LRR2, LRR3, LRR4, LRR6, LRR12, and LRR14 were obviously higher than that of the whole LRR domain (4.4). The MPHA indexes of the motifs LRR5, LRR10, and LRR11 were lower than the average value of the LRR domain, whereas there was no mutation observed in the remaining 7 LRR motifs (Figure 3 and Table 1).

**TABLE 2:** Mutation sites in conserved motifs in the coiled-coil (CC) and NB-ARC domains of PM21 protein.

Motif	Sequence in PM21	Consensus sequence	References
**CC**			
nT motif	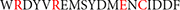	WVxxIRELAYDIEDIVDxY	Bai et al., 2002
**NB-ARC**			
Kinase-1a	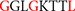	GGLGKTTL	Dilbirligi and Gill, 2003
RNBS-A	FSCKIFFSVSQRP	FDLxAWVCVSQxF	Meyers et al., 1999
Kinase-2	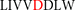	LIVLDDVW	Dilbirligi and Gill, 2003
Kinase-3a	GSRVIVTTR	GSKIIVTTR	Dilbirligi and Gill, 2003
RNBS-C	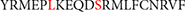	YEVxxLSEDEAWELFCKxAF	Meyers et al., 1999
GLPL	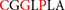	CGGLPLA	Meyers et al., 1999
LPLHLRP*	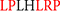	LPx(H/N/Y/D)(L/M/I)(K/R/Q)(T/Q/P)	In this study
RNBS-D	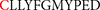	CFLYCALFPED	Meyers et al., 1999
WIAEGF	WVAEGF	WIAEGF	Dilbirligi and Gill, 2003
MHD	VHD	MHD	Dilbirligi and Gill, 2003

The amino acids marked by red were mutation sites found in PM21 protein. *LPLHLRP, a mutation hotspot, was originally reported in this study.

Taken together, these results indicated that the distribution of mutations that led to amino acid changes and loss-of-function of *Pm21* resistance is tendentious in some degrees.

### LPLHLRP immediately close to RNBS-D motif is a new mutation hotspot

**FIGURE 4:**
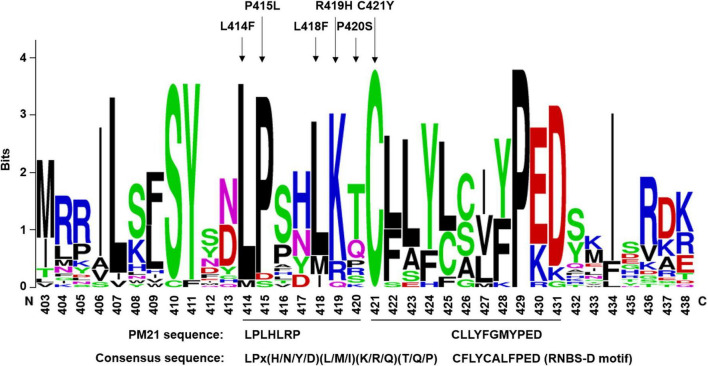
Sequence logos of the mutation hotspot LPLHLRP and its closely linked RNBS-D motif (CFLYCALFPED). Sequence logos were generated from 26 NLR proteins of wheat crops, including PM3, PM8, PM21, LR1, LR21, SR33, SR35, YR10, MLA1, MLA6, and so on. Arrows indicate amino acid changes leading to loss-of-function of PM21. The consensus sequences and the corresponding PM21 sequence are shown at the bottom.

LPLHLRP at position 414–420 in PM21 protein was closely near to the N-terminus of RNBS-D motif in the NB-ARC domain. We observed that 7 susceptible mutants involved in 5 amino acid changes in this region, including L414F (Y18-S12), P415L (Y18-S59), L418F (Y18-S112), R419H (Y18-S58), and P420S (Y18-S19, Y18-S79, and Y18-S82). It meant that the MPHA index of LPLHLRP reached 71.4, significantly higher than those of any other motifs (Table 1). Obviously, LPLHLRP was a mutation hotspot that has not been reported previously. We compared 26 wheat and barley NLR proteins against different diseases and found that the seven amino acids were relatively conserved, with the consensus sequence LPx(H/N/Y/D)(L/M/I)(K/R/Q)(T/Q/P) (Figure 4). It was strongly suggested that this conserved sequence might play an important role(s) in NLR proteins.

### The variant E44K in the coiled-coil domain leads to dominant-negative phenotype

**FIGURE 5:**
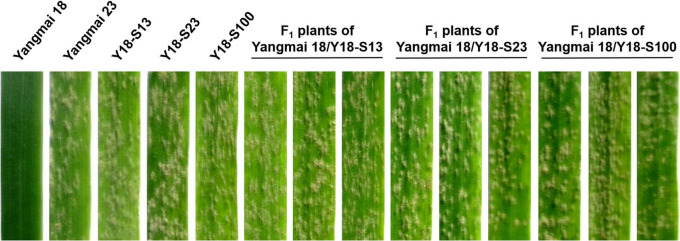
Powdery mildew responses of mutant lines and F_1_ plants involving dominant-negative effect. Responses of the F_1_ plants of the crosses Yangmai 18/Y18-S13, Yangmai 18/Y18-S23, and Yangmai 18/Y18-S100 were tested at one-leaf stage by inoculation with *Bgt* isolate BgtYZ01. Wheat cultivar (cv.) Yangmai 18, Yangmai 23, and the mutant lines Y18-S13, Y18-S23, and Y18-S100 were used as controls.

All susceptible mutants were crossed with Yangmai 18 carrying wild-type *Pm21*, and the powdery mildew responses of the generated F_1_ plants were then assessed. The results showed that all the F_1_ plants derived from three crosses, Yangmai 18/Y18-S13, Yangmai 18/Y18-S23, and Yangmai 18/Y18-S100, were susceptible to BgtYZ01 (Figure 5), whereas all F_1_ plants derived from other crosses were resistant. The susceptible and resistant individuals in the F_2_ plants derived from the above three crosses were 116 and 35 (χ^2^ = 0.267, *p* = 0.605), 145 and 46 (χ^2^ = 0.086, *p* = 0.770), and 138 and 43 (χ^2^ = 0.149, *p* = 0.699), respectively, which were all fit for the theoretical Mendelian segregation ratio 3:1 (Table 3). Interestingly, all the three independent susceptible mutants (Y18-S13, Y18-S23, and Y18-S100) contained the same mutation G to A at position 130, which led to the amino acid change E to K at position 44 in the CC domain. Therefore, it was strongly suggested that the mutation E44K could lead to dominant-negative phenotype.

**TABLE 3:** Genetic data of three crosses showing dominant-negative effect.

Cross	Number of F_1_ plants		Number of F_2_ plants		χ^2^_3:1_ (*P* > 0.01)
	Susceptible	Resistant	Susceptible	Resistant	
Yangmai 18/Y18-S13	28	0	116	35	0.267 (0.605)
Yangmai 18/Y18-S23	35	0	145	46	0.086 (0.770)
Yangmai 18/Y18-S100	22	0	138	43	0.149 (0.699)

## Discussion

*Pm21*, originated from *D. villosum*, a wild relative of wheat, is one of the most valuable powdery mildew resistance genes that provides effective resistance to all tested isolates of *Bgt* (He et al., 2018; Zhang et al., 2019). Our recent research shows that *Pm21* encodes a CC-type NLR protein. Usually, disease resistance conferred by such kind of genes is subjected to be overcome by fast-evolved pathogen races. So, uncovering the molecular characteristic of PM21 protein will be very important for sustainable utilization of *Pm21* in wheat production. In this study, we performed large-scale mutagenesis on *Pm21* to find its key functional site. Nucleotide changes induced by EMS treatment widely occur in a genome, and some changes out of *Pm21*, such as changes in resistance signal components, may also impede *Pm21* resistance in some degrees. To exclude such non-target mutation leading to loss-of-function as possible, all the candidate susceptible mutants were crossed with Y18-S7, which carries a mutated *Pm21* only encoding very small fragment of PM21 protein and shows complete susceptibility at whole growth stages, and perform allelism tests. Finally, 113 stable susceptible mutants, whose variants leading to loss-of-function were considered to be occurred in the target gene *Pm21*, were identified. Among them, 82 independent mutants were involved in 59 different single amino acid substitutions, on which, we focused in this study.

In the CC domain of NLR, a core motif, EDVID motif (consensus sequence: VRELAYDAEDVID), was identified from the nT motif (consensus sequence: WVxxIRELAYDIEDIVDxY) originally predicted in rice (Bai et al., 2002; Rairdan et al., 2008). The EDVID motif, about 110–130 residues N-terminal to the P-loop motif of the NB-ARC domain, is conserved in most NLRs and required for the intramolecular interaction with the NB-ARC domain (Rairdan et al., 2008). Some mutations occurring in the residues in the EDVID motif can lead to lose cell death-inducing activity and disease resistance of potato Rx and barley MLA10 (Rairdan et al., 2008; Maekawa et al., 2011; Bai et al., 2012). In PM21 protein, a sequence, WRDYVREMSYDMENCIDDF, similar to the EDVID motif, was characterized. In total, three variants, R73Q, E80K, and C82Y, could lead to loss-of-function of *Pm21*. Interestingly, our recent work showed that the CC domain containing any of the variants R73Q and E80K keeps the cell-death inducing activity but inhabits the autoactivation of PM21 (D491V) that harbors a mutation in the MHD motif (Gao et al., 2020). In addition, the variant R69K in the nT motif but not in the EDVID motif could also lead to loss-of-function of *Pm21*.

A recent research reported another motif, MADA motif, in the CC domain of NLR. The consensus sequence of MADA motif has been characterized as MADAxVSFxVxKLxxLLxxEx that lies in the N-terminus of the CC domain of ∼20% plant NLRs (Adachi et al., 2019). The MADA motif corresponds to the α1 helix of *Arabidopsis* ZAR1, which is a conformational switch during resistosome activation (Adachi et al., 2019; Wang et al., 2019a,b). Here, no similar sequence of the MADA motif could be found in PM21 protein. However, a mutation hotspot, ATMGAMNPLIGKL (from position 8–20), was observed in the N-terminus of the CC domain. In this hotspot, 7 variants involved in 6 sites could lead to lose *Pm21* resistance. So, it was speculated that this region might play the roles in PM21 resistosome activation.

The variant E44K in PM21 protein could lead to lose the cell death-inducing activity of the CC domain (Gao et al., 2020). Here, we found three independent mutants susceptible to powdery mildew that shows dominant-negative effect when crossed them with Yangmai 18 carrying wild-type *Pm21* gene. Surprisingly, each of the mutants contained the same mutation E44K in PM21 protein. It strongly indicated that E44K is responsible for this dominant-negative effect. In the previous research, two variants involved in the CC domain of *Arabidopsis* RPS2, a CC-NLR, can lead to dominant-negative effect. The first variant R2M1 has a truncated CC domain lacking of position 7–25. The second variant R2M2 has two amino acid substitutions, L38R and T40L (Tao et al., 2000).

Dominant-negative effect of NLR is thought to be caused by defective protein complex which consists of wild-type and mutated NLR (Dinesh-Kumar et al., 2000). Recently, plant NLR resistosome is confirmed to be a functional complex for disease resistance (Wang et al., 2019a,b). Therefore, for dominant-negative effect, one reasonable explanation is that mutated NLR could be integrated into resistosome with wild-type NLR and interfere the normal function of resistosome. In this study, a number of PM21 variants were identified; however, only E44K could cause dominant-negative effect, whereas other variants could not. This phenomenon suggested that most PM21 variants could not disturb the function of resistosome. It was also suggested that, in most situations, resistosome might possess strong fault-tolerance and maintain its resistance function when an abnormal peptide is incorporated into it.

The NB-ARC domain is a molecular switch for regulation of the activity of NLR (Bernoux et al., 2016). It consists of at least nine conserved motifs as reported previously (Meyers et al., 1999; Dilbirligi and Gill, 2003). Among them, the motifs P-loop, Kinase-2, and MHD are well known. P-loop, also called Kinase-1a or Walker A motif, has the consensus sequence GGLGKTTL and makes that NLR has the ability to bind ATP (Meyers et al., 1999; Tameling et al., 2002). Kinase-2, also called Walker B, shares the consensus sequence LIVLDDVW, in which, each aspartate (D) is almost conserved in all plant NLRs. The first D takes part in coordination of the Mg^2+^ ion in the catalytic site, and the second D acts as the catalytic site for ATP hydrolysis and activation of NLR (Meyers et al., 1999; Tameling et al., 2006). MHD lies in the C-terminus of the NB-ARC domain, which is known because the change aspartate (D) to valine (V) can enhance the binding between NLR and ATP and result in the autoactivation of many NLRs (Bendahmane et al., 2002; Tameling et al., 2006; Gao et al., 2020). According to the result of sequence comparison, PM21 protein contains all the nine motifs. Our mutational analysis showed that 10 variants, leading to loss-of-function of PM21, were involved in amino acid substitutions in the core sites of 5 reported motifs of the NB-ARC domain (Table 2).

The sequence of PM21 P-loop motif is GGLGKTTL (position: 196–203), which well matches the consensus sequence of this motif. Mutational analysis showed that each of the six mutations such as G196R, G196E, G197D, G199D, L203F (occurring in P-loop), and G194E (near the N-terminus of P-loop) could lead to loss-of-function of PM21. Mutational research on tobacco N reveals that specific mutations, including G216A/E/V/R, G218R, G219D, K222E/N, and T223A/N, near or in the P-loop not only lead to lose N function but also result in dominant-negative effects (Dinesh-Kumar et al., 2000). However, in another research on *Arabidopsis* RPM1, no dominant-negative effect was detected in the mutant line rpm1-94 that carries the variant G200E corresponding to G216A/E/V/R of N (Tornero et al., 2002). Here, the variants G194E and G196R/E of PM21 corresponded to G216A/E/V/R and G218R of N, respectively. As our observation, all the F_1_ plants derived from the crosses of the corresponding susceptible mutants with Yangmai 18 (harboring wild-type *Pm21*) showed complete resistance to powdery mildew, indicating that these variants could not interfere with PM21 function.

Previously, we found a susceptible natural accession of *D. villosum* that carries a non-functional *Pm21* allele with a transition A821G resulting in the amino acid change D274G in the Kinase-2 (Walker B) motif (He et al., 2020). Here, through EMS treatment, *Pm21* in the susceptible mutants Y18-S28 was identified to be containing a transition G817A resulting in the substitution D273N. The results suggested that the two D residues are important for Kinase-2 function and PM21 resistance, which is in accordance with the previous reports on *Arabidopsis* RPS2 (Tao et al., 2000), tobacco N (Dinesh-Kumar et al., 2000), and tomato I-2 (Tameling et al., 2006).

In the NB-ARC domain, the functions of another six motifs such as RNBS-A, RNBS-B (Kinase-3a), RNBS-C, GLPL, RNBS-D, and WIAEGF are not well known. It is believed that these motifs may play the roles in binding ATP for the regulation of NLR activity (Takken et al., 2006). Mutational analysis of *Arabidopsis* RPM1 shows that three variants, E340K, A341V, and L344F, respectively located in the 10th, 11th, and 14th residues in the RNBS-C motif, could result in RPM1 function defect (Tornero et al., 2002). Here, we demonstrated that two variants, L324F and S329L, occurred in the first and 11th residues in the RNBS-C motif could lead to lose PM21 resistance. S329L of PM21 and A341V of RPM1 situate in the same position of the RNBS-C motif, whereas L324F of PM21 is a new found functional site.

The GLPL motif has a conserved sequence CGGLPLA. Mutational analyses of flax rust resistance protein P2 (variant: G395E) (Dodds et al., 2001), maize Rp1-D21 (variant: P398L) (Wang et al., 2005), and RPM1 (variant: A379V) (Tornero et al., 2002) indicate the importance of the 3rd residue glycine (G), the 5th residue proline (P) and the last residue alanine (A). In this study, we obtained six independent susceptible mutants involved in four variants of three sites (C359Y, G361R, P363S, and P363L) in the GLPL motif. It was suggested that not only the 3rd residue G and the 5th residue P but also the first residues cysteine (C) are crucial for PM21 resistance.

The RNBS-D motif has a consensus sequence CFLYCALFPED. RPM1 variants (S439F and P442L) at the 6th and 9th residues in this motif result in loss-of-function (Tornero et al., 2002). Here, we revealed that replacement of the first residue cysteine (C) with tyrosine (Y) (C421Y) could lead to hampering PM21 resistance, indicating the importance of the cysteine residues in NLR. Interestingly, near the N-terminus of the RNBS-D motif, seven independent mutations, involved in five sites (L414F, P415L, L418F, R419H, and P420S), were observed in this study, suggesting that the sequence LPLHLRP of PM21 seems to be a mutation hotspot leading to loss-of-function. Comparative analysis showed that both the LPLHLRP-like sequences and the RNBS-D sequences are conserved in different NLRs. Due to that LPLHLRP is immediate before the reported motif RNBS-D, we propose that it should be considered as an extended functional region of the RNBS-D motif rather than as a new independent motif.

The LRR domain is more variant than other domains in NLR, which is usually considered as a sensor for specific recognition of pathogen effector by a direct or indirect means (Dangl et al., 2013). This is supported by several researches. For instance, a single amino acid difference can make that rice blast resistance gene *Pi-ta* differs from its susceptible allele (Bryan et al., 2000). A total of six amino acid changes can alter flax rust resistance gene *P2* to *P* specificities (Dodds et al., 2001). Natural variations in the LRR domain result in different powdery mildew responses of *Pm3* alleles (Srichumpa et al., 2005). A recent research showed that site-directed mutagenesis on the LRR domain of PM3A can obviously control response strength and resistance spectrum (Lindner et al., 2020). In this research, a total of 25 susceptible mutants involved in 17 amino acid changes were obtained. In our recent work, the LRR domain of PM21 has been divided into 16 LRR motif with the consensus sequence LxxLxLxx (where L represents a conserved leucine or other aliphatic residue and x represents any amino acid) (He et al., 2020). These variants were distributed in 9 LRR motifs (LRR2–LRR6, LRR10–LRR12, and LRR14), some of which have been confirmed to be under diversifying selection (He et al., 2020). Of 17 variants, 2 (L571F and L787F), 4 (R551H, E624K, P654S, and E839K), and other 11 occurred in the position of L, x, and the non-core region of LRR motif, respectively. It was suggested the importance of these sites for complete PM21 resistance. Whether the amino acid changes in the LRR domain alter recognition specificity for *Bgt* isolates needs further investigation.

In conclusion, we performed large-scale mutagenesis on wheat powdery mildew resistance gene *Pm21*, which revealed the distribution characteristics of variant amino acid residues and discussed their potential impacts on PM21 resistance. To our knowledge, some mutation sites have not been reported in plant NLRs previously. Furthermore, two mutation hotspots, one near the N-terminus of the CC domain and the other immediately close to the N-terminus of RNBS-D motif of the NB-ARC domain and a variant resulting in dominant-negative effect, were identified. This study will contribute to understanding the relationship of resistance and structure of *Pm21*-encoded NLR, which would be useful for the development of durable resistance for efficiently controlling wheat powdery mildew.

## Data availability statement

The datasets presented in this study can be found in online repositories. The names of the repository/repositories and accession number(s) can be found in the article/Supplementary material.

## Author contributions

HH and SZ conceived and designed the experiments and wrote and revised the manuscript. HH, RG, ZC, RL, and XK screened the mutants and identified the mutation sites. HH performed allelism test. HH, AG, and TL analyzed the data. All authors contributed to the article and approved the submitted version.
