# Supplementary material for: Large-scale mutational analysis of wheat powdery mildew resistance gene Pm21
Source: Front Plant Sci. 2022 Aug 9;13:988641. doi: 10.3389/fpls.2022.988641 (PMC9396339; doi:10.3389/fpls.2022.988641)
Supplement: Supplementary file 1 [file Table_1.DOCX]

**Table S1 Mutations of *Pm21* in the susceptible Yangmai 18 mutants. Asterisks indicate premature stop codons.**

| **Mutant** | **Mutation in cDNA** | **Amino acid change** | **Domain or motif** |
| --- | --- | --- | --- |
| Single base mutation changing amino acid | | | |
| Y18S41 | C23T | A8V | CC |
| Y18S77 | C26T | T9I | CC |
| Y18S94 | G32A | G11E | CC |
| Y18S10 | C43T | P15S | CC |
| Y18S105 | C44T | P15L | CC |
| Y18S104 | G53A | G18D | CC |
| Y18S73 | C58T | L20F | CC |
| Y18S13 | G130A | E44K | CC |
| Y18S23 | G130A | E44K | CC |
| Y18S100 | G130A | E44K | CC |
| Y18S60 | C140T | A47V | CC |
| Y18S96 | C188T | P63L | CC |
| Y18S107 | G206A | R69K | nT motif of CC |
| Y18S55 | G218A | R73Q | nT motif of CC |
| Y18S83 | G218A | R73Q | nT motif of CC |
| Y18S34 | G238A | E80K | nT motif of CC |
| Y18S102 | G238A | E80K | nT motif of CC |
| Y18S44 | G245A | C82Y | nT motif of CC |
| Y18S37 | G349A | A117T | CC |
| Y18S103 | G349A | A117T | CC |
| Y18S17 | G401A | R134H | CC |
| Y18S1 | G503A | G168D | Linker between CC and NB-ARC |
| Y18S5 | G503A | G168D | Linker between CC and NB-ARC |
| Y18S18 | G503A | G168D | Linker between CC and NB-ARC |
| Y18-S111 | C526T | L176F | Before Kinase-1a (P-loop) of NB-ARC |
| Y18S81 | C572T | A191V | Before Kinase-1a (P-loop) of NB-ARC |
| Y18S15 | G581A | G194E | Before Kinase-1a (P-loop) of NB-ARC |
| Y18S68 | G581A | G194E | Before Kinase-1a (P-loop) of NB-ARC |
| Y18S2 | G586A | G196R | Kinase-1a (P-loop) of NB-ARC |
| Y18S11 | G587A | G196E | Kinase-1a (P-loop) of NB-ARC |
| Y18S86 | G587A | G196E | Kinase-1a (P-loop) of NB-ARC |
| Y18S69 | G590A | G197D | Kinase-1a (P-loop) of NB-ARC |
| Y18S31 | G596A | G199D | Kinase-1a (P-loop) of NB-ARC |
| Y18S26 | C607T | L203F | Kinase-1a (P-loop) of NB-ARC |
| Y18S28 | G817A | D273N | Kinase-2 of NB-ARC |
| Y18S84 | G913A | D305N | After Kinase-3a (RNBS-B) of NB-ARC |
| Y18S74 | G919A | A307T | After Kinase-3a (RNBS-B) of NB-ARC |
| Y18S38 | C970T | L324F | RNBS-C of NB-ARC |
| Y18S14 | C986T | S329L | RNBS-C of NB-ARC |
| Table S1 (continued) | | | |
| **Mutant** | **Mutation in cDNA** | **Amino acid change** | **Domain or motif** |
| Y18S22 | G1076A | C359Y | GLPL of NB-ARC |
| Y18S48 | G1081A | G361R | GLPL of NB-ARC |
| Y18S52 | C1087T | P363S | GLPL of NB-ARC |
| Y18S24 | C1088T | P363L | GLPL of NB-ARC |
| Y18S49 | C1088T | P363L | GLPL of NB-ARC |
| Y18S50 | C1088T | P363L | GLPL of NB-ARC |
| Y18S12 | C1240T | L414F | Before RNBS-D of NB-ARC |
| Y18S59 | C1244T | P415L | Before RNBS-D of NB-ARC |
| Y18S112 | C1252T | L418F | Before RNBS-D of NB-ARC |
| Y18S58 | G1256A | R419H | Before RNBS-D of NB-ARC |
| Y18S19 | C1258T | P420S | Before RNBS-D of NB-ARC |
| Y18S79 | C1258T | P420S | Before RNBS-D of NB-ARC |
| Y18S82 | C1258T | P420S | Before RNBS-D of NB-ARC |
| Y18S65 | G1262A | C421Y | Before RNBS-D of NB-ARC |
| Y18S25 | G1312A | D438N | Between RNBS-D and WIAEGF of NB-ARC |
| Y18S70 | G1384A | E462K | Between WIAEGF and MHD of NB-ARC |
| Y18S27 | G1528A | A510T | Linker between NB-ARC and LRR |
| Y18S92 | G1528A | A510T | Linker between NB-ARC and LRR |
| Y18S9 | G1652A | R551H | In the 2nd LRR |
| Y18S39 | G1670A | G557E | In the 2nd LRR |
| Y18S64 | G1670A | G557E | In the 2nd LRR |
| Y18S32 | C1711T | L571F | LRVLDL motif in the 3rd LRR |
| Y18S72 | C1711T | L571F | LRVLDL motif in the 3rd LRR |
| Y18S89 | C1766T | T589I | In the 3rd LRR |
| Y18S75 | G1775A | G592D | In the 3rd LRR |
| Y18S109 | C1831T | P611S | In the 4rd LRR |
| Y18S99 | C1849T | L617F | In the 4rd LRR |
| Y18S20 | G1870A | E624K | In the 5th LRR |
| Y18S101 | G1870A | E624K | In the 5th LRR |
| Y18S106 | G1870A | E624K | In the 5th LRR |
| Y18S93 | C1942T | P648S | In the 6th LRR |
| Y18S63 | C1954T | R652C | In the 6th LRR |
| Y18S3 | C1960T | P654S | In the 6th LRR |
| Y18S76 | C1960T | P654S | In the 6th LRR |
| Y18S85 | C2261T | P754L | In the 10th LRR |
| Y18S54 | G2344A | G782R | In the 11th LRR |
| Y18S45 | C2359T | L787F | In the 12th LRR |
| Y18S108 | C2359T | L787F | In the 12th LRR |
| Y18S40 | G2396A | G799E | In the 12th LRR |
| Y18S87 | G2515A | E839K | In the 14th LRR |
| Y18S30 | C2546T | P849L | In the 14th LRR |
| Y18S95 | C2546T | P849L | In the 14th LRR |
| Table S1 (continued) | | | |
| **Mutant** | **Mutation in cDNA** | **Amino acid change** | **Domain or motif** |
| Y18S98 | C2546T | P849L | In the 14th LRR |
|  | | | |
| Single base mutation leading to premature stop | | | |
| Y18S36 | G537A | W179* |  |
| Y18S57 | G537A | W179* |  |
| Y18S56 | C640T | Q214* |  |
| Y18S51 | C757T | Q253* |  |
| Y18S42 | C832T | Q278* |  |
| Y18S62 | C832T | Q278* |  |
| Y18S97 | C832T | Q278* |  |
| Y18S7 | G843A | W281* |  |
| Y18S78 | G843A | W281* |  |
| Y18S21 | G926A | W309* |  |
| Y18S71 | G926A | W309* |  |
| Y18S53 | C979T | Q327* |  |
| Y18S113 | A1069T | K357* |  |
| Y18S4 | C1129T | Q377* |  |
| Y18S88 | G1148A | W383* |  |
| Y18S16 | G1149A | W383* |  |
| Y18S33 | G1328A | W443* |  |
| Y18S67 | G1328A | W443* |  |
| Y18S61 | G1328A | W443* |  |
| Y18S46 | G1329A | W443* |  |
| Y18S29 | C1645T | Q549* |  |
| Y18S91 | G2268A | W756* |  |
| Y18S8 | C2500T | R834* |  |
| Y18S110 | C2500T | R834* |  |
| Y18S47 | G2532A | W844* |  |
|  | | | |
| Two-base mutation | | | |
| Y18-S35 | G265A | D89N | CC |
|  | G927A | W309* |  |
| Y18-S43 | G464A | R155Q | Linker between CC and NB-ARC |
|  | G1775A | G592D | In the 3rd LRR |
| Y18-S66 | G22A | A8T | CC |
|  | G2026A | D676N | In the 7th LRR |
| Y18-S80 | G589A | G197S | Kinase-1a (P-loop) of NB-ARC |
|  | G2490A | M830I | In the 13th LRR |
| Y18-S90 | G511A | G171S | Linker between CC and NB-ARC |
|  | G1329A | W443* |  |
|  |  |  |  |
|  |  |  |  |
| Table S1 (continued) | | | |
| Fragment deletion | | | |
| Y18-S6 | The deleted region was flanked by markers 6VS-03 and 6VS-10.2 (He et al., 2017). | | |

The susceptible mutant lines Y18-S1 ~ Y18-S58 were obtained in our previous work (He et al., 2018) and the others were newly identified in this study.
